# *Salvia miltiorrhiza* Root Extract as a Potential Therapeutic Agent for IgE/Ag-Induced Allergic Reactions and Atopic Dermatitis via the Syk/MAPK Pathway

**DOI:** 10.3390/biomedicines13071547

**Published:** 2025-06-25

**Authors:** Min-ah Kim, Jin-Ho Lee, Keunjung Woo, Eunwoo Jeong, Tack-Joong Kim

**Affiliations:** 1 Division of Biological Science and Technology, Yonsei University, Wonju 26493, Republic of Korea; mina1218@yonsei.ac.kr (M.-a.K.); drlogos@naver.com (J.-H.L.); rmswjd@yonsei.ac.kr (K.W.); jew0108@naver.com (E.J.); 2 Research & Development Center, Doctor TJ Co., Ltd., Wonju 26493, Republic of Korea

**Keywords:** *Salvia miltiorrhiza*, allergy, anaphylaxis, atopic dermatitis

## Abstract

**Background/Objectives**: Allergens can trigger severe immune responses in hypersensitive individuals, with mast cells releasing inflammatory mediators via IgE-FcɛRI signaling. Spleen tyrosine kinase (Syk) is a key regulator in this pathway, making it a promising therapeutic target. Natural modulators of Syk-mediated mast cell activation remain underexplored. This study investigated the anti-allergic effects of a 70% ethanol extract of *Salvia miltiorrhiza* (SME) using in vitro and in vivo models. **Methods**: SME was evaluated using IgE-sensitized RBL-2H3 cells, a passive cutaneous anaphylaxis model, and a DNCB-induced atopic dermatitis-like mouse model. Allergic responses were assessed via degranulation assays, histopathology, serum IgE levels, and the spleen index. **Results**: SME significantly inhibited mast cell degranulation by 44.4 ± 1.6% in RBL-2H3 cells at 100 µg/mL following 30 min of treatment compared to the untreated control. Western blot analysis demonstrated dose-dependent suppression of protein kinase B (PKB, also known as AKT), c-Jun N-terminal kinase (JNK), extracellular signal-regulated kinase (ERK), and spleen tyrosine kinase (Syk) phosphorylation, indicating inhibition of key allergic signaling pathways. In an IgE/Ag-induced passive cutaneous anaphylaxis model in ICR mice, SME (100 mg/kg, orally) significantly attenuated vascular permeability, as evidenced by a 20.6 ± 9.7% reduction in Evans blue extravasation relative to the Ag-treated group. In a 1-chloro-2,4-dinitrobenzene (DNCB)-induced atopic dermatitis (AD)-like model, six treatments of SME significantly improved the skin condition, reduced spleen enlargement associated with allergic inflammation, and decreased serum IgE levels by 43.3 ± 11.2% compared to the DNCB group. **Conclusions**: These findings suggest that SME may help to alleviate allergic responses and AD by modulating key immune signaling pathways.

## 1. Introduction

Allergens are harmless substances for most individuals; however, they can trigger allergic reactions in hypersensitive individuals. These reactions include urticaria, asthma, rhinitis, and severe cases, anaphylaxis. Common allergens include pollen, animal dander, dust, and specific foods [1]. Moreover, chemical air pollutants, such as PM and ozone, can interact with these allergens, amplifying their allergic potential and contributing to the increased prevalence of allergies [2]. Among these, anaphylaxis is particularly dangerous because it involves simultaneous multi-organ symptoms, such as neurological dysfunction and hypotension, which can be life-threatening without immediate medical intervention [3]. Mast cells play a central role as effectors in allergic immune responses. They are widely distributed in tissues exposed to the external environment, including the skin, mucous membranes, nerves, and blood vessels [4]. Mast cells express high-affinity IgE receptors (FcɛRI) that bind allergen-specific IgE. Upon receptor aggregation, mast cells undergo degranulation, releasing inflammatory mediators, such as histamine, leukotrienes, and prostaglandins, which cause allergic inflammation [5,6]. Although mast cells are known for their role in acute allergic reactions, recent studies have suggested that they contribute to innate immune responses. Mast cells use pattern recognition receptors to detect pathogens and modulate immune activation [7,8]. However, IgE-mediated signaling remains the dominant mechanism in immediate-type allergic reactions [9,10]. Individuals with asthma, atopic dermatitis (AD), and allergic rhinitis often exhibit considerably elevated IgE levels, particularly in the lungs, skin, and mucosal tissues [11].

AD is one of the most prevalent chronic allergic conditions globally. Pruritogenic mediators derived from mast cells trigger persistent itching, leading to repeated scratching, further skin barrier damage, and exacerbated inflammation [12,13]. Spleen tyrosine kinase (Syk), as a key regulator of allergic inflammation, mediates immunoreceptor signaling in various immune cells, including B cells, mast cells, macrophages, and neutrophils [14]. Syk inhibition effectively suppresses mast cell degranulation and mediator release, blocking the release of preformed mediators (e.g., histamine), lipid mediators (e.g., leukotrienes and prostaglandins), and cytokines. As an upstream signaling molecule, Syk is a more effective therapeutic target than downstream effectors [15,16]. Additionally, Fc receptors (FcRs) play a crucial role in regulating the activation thresholds of immune cells in antibody-mediated immune responses, highlighting their significance in allergic reactions [17]. As Syk and MAPK signaling are key pathways in allergic responses, identifying natural compounds that modulate these pathways may provide novel therapeutic options. This study investigates *Salvia miltiorrhiza* as a potential mast cell inhibitor [18,19].

*S. miltiorrhiza*, a medicinal plant traditionally used for cardiovascular treatments, has antioxidant, neuroprotective, anti-inflammatory, and anti-angiogenic properties [20]. It is primarily cultivated in central and eastern China, including Henan, Shanxi, Sichuan, and Anhui provinces, where soil conditions and cultivation practices significantly influence its growth and the profile of bioactive compounds [21]. Its major active constituents, such as tanshinones and salvianolic acids, are biosynthesized through tightly regulated metabolic pathways and are known for their immunomodulatory and anti-inflammatory activities [22]. Therefore, this study aimed to explore the potential of *S. miltiorrhiza* as a novel therapeutic agent for allergic inflammation by investigating its role in mast cell inhibition and Syk signaling regulation [23,24].

## 2. Materials and Methods

### 2.1. Reagents

2,4-Dinitrophenylated-conjugated bovine serum albumin (DNP-BSA) was purchased from Thermo Fisher (Waltham, MA, USA). Olive oil, diphenylhydramine (DPH), 1-chloro-2,4-dinitrobenzene (DNCB), penicillin streptomycin, trypsin-EDTA, mouse monoclonal anti-DNP IgE antibody, and Evans blue were purchased from Sigma-Aldrich (St. Louis, MO, USA). The Mouse IgE ELISA Deluxe Set was acquired from BioLegend (San Diego, CA, USA). The QuantiMAX™ WST-8 Cell Viability Assay Kit was purchased from BIOMAX (Seoul, Republic of Korea). Fetal bovine serum was sourced from Access Biological (Vista, CA, USA).

### 2.2. Extraction Preparation of S. miltiorrhiza Extract

*Salvia miltiorrhiza* was cultivated following the Good Agricultural Practices (GAP) guidelines of the Korea Rural Development Administration and harvested in 2020 in Eumseong, Korea (GPS: E 128°62′, N 36°56′). For sample preparation, the roots of *S. miltiorrhiza* were roasted at 240 °C for 10 min and extracted three times with 70% ethanol at 85 °C for 2 h. The extracts were filtered through Whatman No. 1 filter paper, combined, and concentrated using a rotary evaporator (EYELA N-1000, Tokyo Rikakikai Co., Ltd., Tokyo, Japan) at 40 °C. The concentrated extract was subsequently lyophilized and powdered (Serial No: CR10240). The extraction yield was 26.31% (*w*/*w*).

### 2.3. Cell Culture

The RBL-2H3 cell line was purchased from the American Type Culture Collection (Rockville, MD, USA). Cells were cultured in Eagle’s minimal essential medium (WelGENE, Inc., Daegu, Republic of Korea) containing 10% (*v*/*v*) fetal bovine serum, 100 U/mL penicillin, and 100 U/mL streptomycin at 37 °C in a humidified atmosphere of 95% air and 5% CO_2_.

### 2.4. Cytotoxicity Assay

Cytotoxicity was determined using the Quanti-Max WST-8 cell viability kit. RBL-2H3 cells were seeded at 1 × 10^5^ cells/mL in 96-well plates and incubated for 24 h. After 24 h, the medium was removed and the cells were treated with 0–100 µg/mL SME for another 24 h. Then, the extract was removed, and MEM containing 10% Quanti-Max reagent was added to each well and incubated for 30 min at 37 °C. Absorbance was measured at 450 nm using a microplate reader (BioTek Instruments Inc., Winooski, VT, USA).

### 2.5. β-Hexosaminidase Release Assay

RBL-2H3 cells were seeded at a density of 4 × 10^5^ cells/mL in 24-well plates and incubated at 37 °C for 12 h. The cells were sensitized with DNP-specific IgE (200 ng/mL) and incubated for 12 h. The plates were washed with Siraganian buffer (pH 7.2, 120 mM NaCl, 5 mM KCl, 0.85 mM MgCl2, 25 mM PIPES, 40 mM NaOH) and treated with various concentrations of *S. miltiorrhiza* extract (SME) (0, 25, 50, and 100 µg/mL) for 30 min. DNP-BSA antigen (50 ng/mL) was added and the cells were incubated at 37 °C for 15 min. The reaction was terminated by placing the mixture on ice. The supernatant was centrifuged (200× *g*, 3 min) and transferred to 96-well plates. β-hexosaminidase substrate (1 mM p-NAG) was added in 0.1 M citrate buffer (pH 4.5) and the mixture was incubated at 37 °C for 1 h. The reaction was terminated by adding 0.1 M carbonate buffer (pH 10.5). Absorbance was measured at 405 nm using a FLx800 microplate reader (BioTek Instruments Inc., Winooski, VT, USA) [25].

### 2.6. Passive Cutaneous Anaphylaxis

DNP-specific IgE antibody (0.5 µg per mouse) was injected into one ear of 7-week-old ICR mice. After 24 h, the mice were orally administered SME. DNP-BSA containing 3% Evans blue (200 µL) was intravenously injected an hour later. After 1 h, Ag-challenged mice were euthanized, and their ears were transferred to a 24-well plate. Thereafter, 500 µL of formamide was added, and the plate was incubated at 63 °C for 12 h to extract the dye. The amount of dye was quantified by measuring absorbance at 650 nm using a microplate reader [26].

### 2.7. Immunoblotting and Antibodies

RBL-2H3 cells were seeded at a density of 4 × 10^5^ cells/mL in 6-well plates and incubated for 12 h. The cells were sensitized with DNP-specific IgE (200 ng/mL) and incubated for 12 h. Thereafter, the cells were treated with various concentrations of SME (0, 25, 50, and 100 µg/mL) for 30 min, the antigen (DNP-BSA, 50 ng/mL) was added, and the cells were incubated at 37 °C for 9 min. The reaction was terminated by placing the mixture on ice. After removing the media, lysis buffer was added to each well. The cells were harvested and analyzed using sodium dodecyl sulfate-polyacrylamide gel electrophoresis on 12.5% polyacrylamide gels, and the proteins were transferred to polyvinylidene difluoride membranes. The membranes were subsequently washed and blocked. Thereafter, the membranes were incubated with primary antibodies (1:2500 dilution) at 4 °C overnight on a shaker. After incubation, the membranes were washed, and secondary antibodies (1:5000 dilution) were added and incubated at 26 °C for 2 h on a shaker. Proteins were detected using Chemiluminescent Plus detection reagent for western blotting on the Image Quant LAS 4000 system (GE Healthcare, Buckinghamshire, UK). The antibodies used in the study are as follows: β-actin (4967S), Syk (12358S), p-Syk (2710S), ERK (4695S), p-ERK (4377S), JNK (9252S), p-JNK (9251S), AKT (9272S), and p-AKT (9271S; Cell Signaling Technology, Danvers, MA, USA).

### 2.8. AD-like Mice Model

ICR mice were divided into three groups (control, AD-like model, and SME treatment; n = 6 per group). One day after dorsal hair removal, 250 µL of 1% DNCB solution (dissolved in a mixture of acetone:olive oil = 3:1) was applied to a 7.5 cm^2^ area of dorsal skin and 30 µL of 0.5% DNCB solution was applied to the ears for four consecutive days. After sensitization, 200 µL of 0.5% DNCB solution was applied to the dorsal skin and 30 µL to the ears every 2 d for 2 weeks. Thereafter, 200 µL of 10 mg/mL SME was added to the DNCB-induced dorsal skin and 30 µL of 10 mg/mL SME was applied to the ears six times over 2 weeks. The mice were euthanized 18 d after the first DNCB application. The spleen was dissected and measured, the dorsal skin was isolated for histopathological experiments, and blood was collected from the heart to determine serum IgE levels [27].

### 2.9. Evaluation of Skin Dermatitis Severity

The skin severity score of dermatitis in the dorsal skin was evaluated after treatment with SME. The evaluated symptoms, including (1) redness, (2) crusts/oozing, (3) traces of scratching, (4) erosion, and (5) lichenification, were scored as follows: 0, no symptoms; 1, mild symptoms; 2, moderate symptoms; and 3, severe symptoms. The sum of the scores for the evaluated symptoms (maximum score = 15) was considered the skin severity score.

### 2.10. Ear Condition in AD-like Mice Model

After the last treatment with SME, the ear vascular permeability level was evaluated based on symptoms such as redness and edema.

### 2.11. Histopathological Observation of Skin Tissues

The dorsal skin was isolated from each mouse and fixed in 4% paraformaldehyde solution. The fixed skin tissue was embedded in paraffin, sectioned using a microtome, and stained with hematoxylin and eosin. Histological analysis was performed using a light microscope (NIKON ECLIPSE TS100, Nikon Corporation, Tokyo, Japan). Dorsal skin thickness was measured using a micrometer.

### 2.12. Measuring Spleen Weight

After euthanasia, the body weight of each mouse was measured. The spleens were isolated and weighed. The spleen index value was calculated using the following formula: spleen index = spleen weight (mg)/body weight (g).

### 2.13. Determination of Serum IgE Level

Serum IgE levels were measured using an ELISA MAX™ Deluxe Set Mouse IgE kit (BioLegend, San Diego, CA, USA), according to the manufacturer’s instructions.

### 2.14. Statistical Analyses

Experimental results are expressed as the mean ± standard error of the mean. One-way or two-way analysis of variance and *t*-tests were used. Statistical significance was set at *p* < 0.05.

## 3. Results

### 3.1. SME Suppressed Mast Cell Degranulation

**Figure 1:**
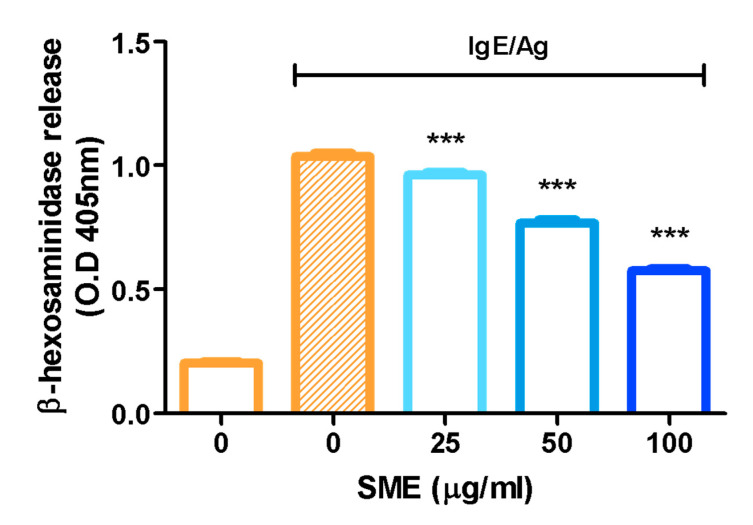
Effect of SME on mast cell degranulation. RBL-2H3 cells were treated with various concentrations of SME (0, 25, 50, and 100 µg/mL), and the cells were challenged with DNP-BSA (50 ng/mL). β-Hexosaminidase release was determined by measuring the absorbance at 405 nm using a microplate reader. Data are expressed as the mean ± SD (n = 6). *** *p* < 0.001.

β-Hexosaminidase, an enzyme stored in the secretory granules of mast cells, is co-released with histamine during immunological reactions. Therefore, β-hexosaminidase is widely recognized as a key marker for assessing mast cell degranulation. To evaluate the anti-allergic effects of SME, β-hexosaminidase release was measured as an indicator of mast cell degranulation. The results demonstrated that SME treatment significantly reduced β-hexosaminidase release, suggesting that SME effectively suppresses mast cell degranulation and allergic mediator secretion (Figure 1). Specifically, SME did not induce cytotoxicity in RBL-2H3 cells at concentrations up to 100 µg/mL, which were used in the β-hexosaminidase release assay (Figure S1).

### 3.2. SME Suppressed IgE/Ag-Induced Passive Cutaneous Anaphylaxis (PCA) In Vivo

**Figure 2:**
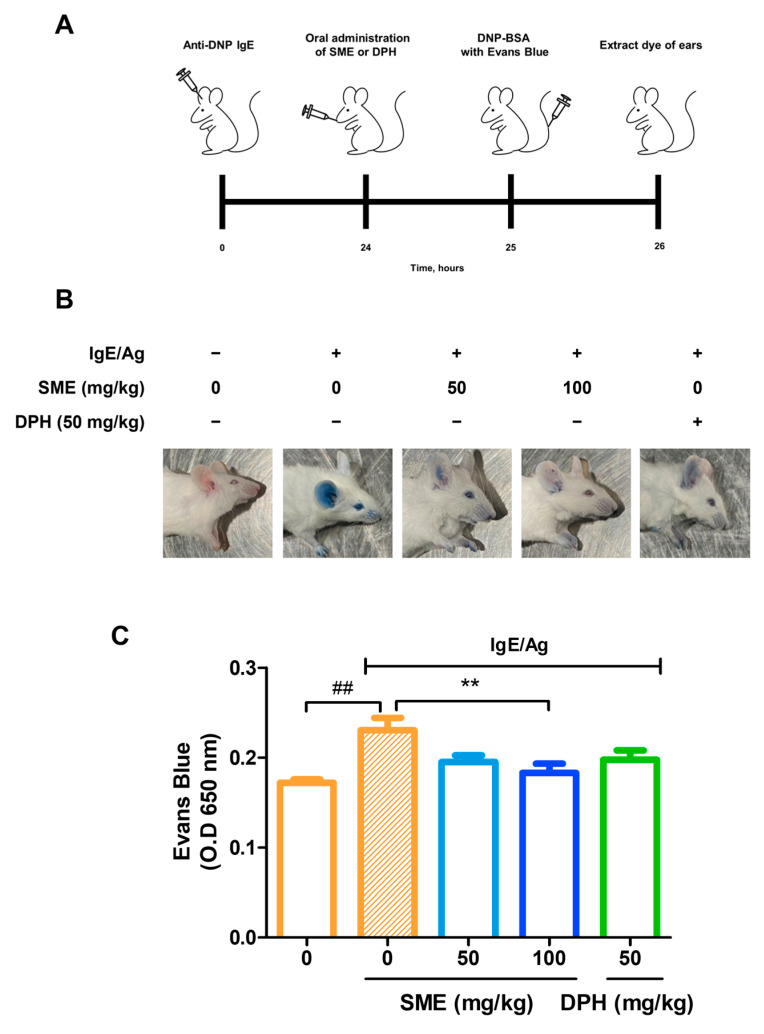
SME suppresses IgE/Ag-induced passive cutaneous anaphylaxis (PCA) in vivo. Seven-week-old ICR mice were injected intradermally with DNP-specific IgE (0.5 µg) into one ear and, after 24 h, were orally challenged with SME (0, 0, 50, 100 mg/kg) or DPH (50 mg/kg). One hour after, DNP-BSA containing 3% Evans blue was intravenously injected. The figure includes a schematic diagram (**A**), representative images of mouse ears (**B**), and quantitative analysis of Evans blue extravasation (**C**). Evans blue was extracted from ear tissue using formamide, and the amount was quantified by measuring the absorbance. Data are expressed as the mean ± SD (n = 5). ^##^  *p* < 0.01 and ** *p* < 0.01.

PCA is a well-established in vivo model for evaluating local allergic reactions mediated by mast cell activation and IgE-dependent hypersensitivity. This model is widely used to assess vascular permeability changes induced by anaphylactic responses. To investigate the anti-allergic effects of SME, IgE was intradermally injected into the ears of mice. After 24 h, various concentrations of SME (0, 50, and 100 mg/kg) were orally administered, and the results were compared with those of DPH, a standard antihistamine. Subsequently, antigen (Ag) mixed with Evans blue dye was injected into the tail vein, and vascular permeability was assessed by quantifying Evans blue extravasation at an absorbance of 650 nm. Our findings revealed that SME treatment significantly reduced Evans blue leakage in a dose-dependent manner (Figure 2B,C). This suggests that SME effectively inhibits mast cell-mediated increases in vascular permeability associated with anaphylaxis. Thus, SME is a potential natural anti-allergic agent capable of modulating IgE/Ag-induced hypersensitivity reactions.

### 3.3. SME Inhibited Phosphorylation of Allergic Response-Related Signaling Proteins

**Figure 3:**
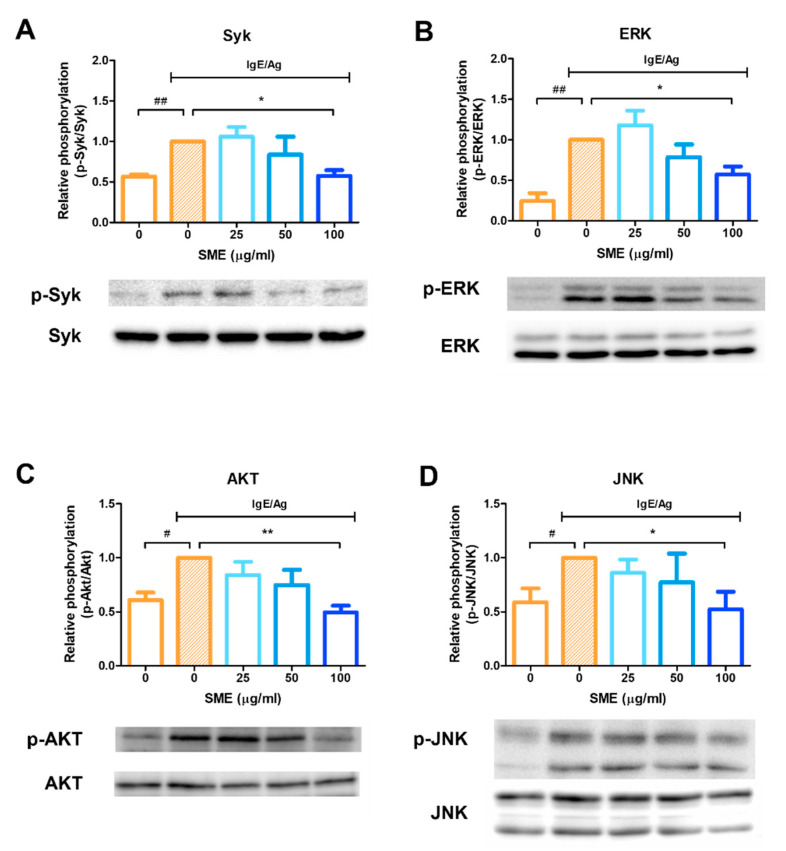
SME inhibits phosphorylation of allergic response-related signaling proteins. RBL-2H3 cells were seeded and incubated in 6-well plates, then sensitized with 200 ng/mL of DNP-specific IgE and treated with various concentration of SME (0, 0, 25, 50, and 100 µg/mL). Effect of SME on relative phosphorylation of Syk (**A**), ERK (**B**), Akt (**C**), and JNK (**D**). Data are expressed as the mean ± SD (n = 3). ^#^  *p* < 0.05, ^##^ *p* < 0.01, * *p* < 0.05 and ** *p* < 0.01.

To evaluate the molecular mechanisms underlying the anti-allergic effects of SME, the phosphorylation status of key signaling proteins was analyzed via western blotting. Mast cell activation leads to the release of various mediators, including histamine, eicosanoids, and cytokines, which can be effectively blocked by Syk kinase inhibitors. Therefore, Syk kinase is a critical target for controlling acute and chronic allergic inflammation. Our results demonstrated that SME treatment (100 µg/mL) significantly inhibited Syk phosphorylation (Figure 3A), indicating that SME suppressed the upstream signaling cascade involved in mast cell activation. As ERK inhibition is known to reduce histamine release and decrease IL-5 and IL-13 secretion in mast cells, ERK phosphorylation was further analyzed. SME treatment inhibited ERK phosphorylation (Figure 3B), suggesting that SME modulated IgE/Ag-induced allergic mediator release via ERK suppression. Additionally, AKT and JNK pathways have been implicated in cytokine production in mast cells during allergic responses. Our findings confirmed that SME treatment suppressed AKT and JNK phosphorylation (Figure 3C,D), further supporting its potential role in modulating allergic inflammatory responses. These results indicated that SME inhibits IgE/Ag-induced mast cell activation by suppressing the Syk-mediated signaling pathway [18,24] and possibly interfering with downstream ERK, AKT, and JNK signaling pathways, thereby reducing histamine release, cytokine secretion, and overall allergic inflammation.

### 3.4. SME Improved Skin Condition and Reduced AD Symptoms in an AD-like Model

**Figure 4:**
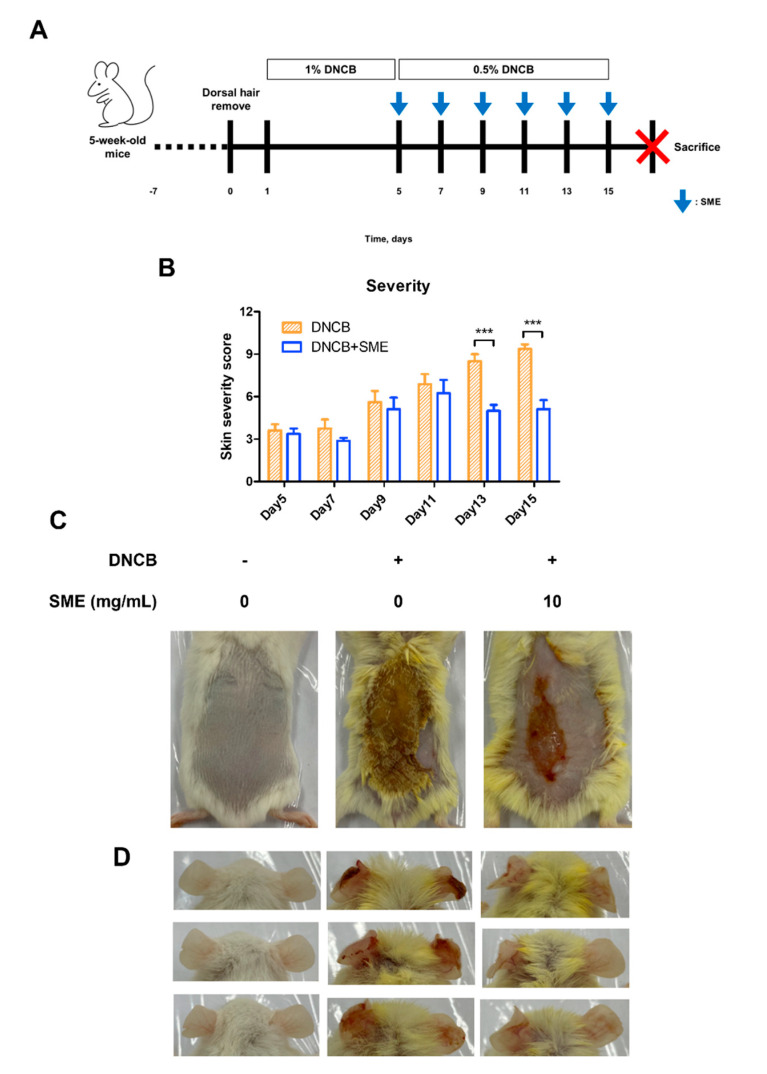
SME improves skin condition and reduces atopic dermatitis symptoms in an AD-like model. During the challenge with atopic dermatitis, SME (10 mg/mL) was treated on the dorsal skin every 2 days, for a total 6 times of 2 weeks. The pictures show the dorsal skin after sacrifice. Control, DNCB (0.5% DNCB), and SME (0.5% DNCB + SME concentration, 10 mg/mL) (**A**). Severity scores were assessed based on erythema, edema, crusting, and lichenification, with scores ranging from 0 (no symptoms) to 3 (severe symptoms). An individual’s score was calculated as the sum of the symptom scores (**B**). Data are expressed as the mean ± SD (n = 8). *** *p* < 0.001. The pictures show the dorsal skin (**C**) and ear (**D**) after sacrifice.

Repeated application of DNCB on mouse dorsal skin is a well-established method for inducing AD-like symptoms, including erythema, crust formation, exudation, scratch marks, erosion, and lichenification (Figure 4A). These symptoms mimic chronic inflammatory skin conditions observed in human AD, making this model suitable for evaluating potential therapeutic agents. To assess the therapeutic effects of SME, skin severity scores were evaluated every 2 d following the initial SME treatment. In the DNCB-only group, the skin severity scores progressively increased due to repeated DNCB applications. However, in the SME treatment group, the severity score decreased after the fifth treatment, indicating progressive skin recovery (Figure 4B,C). In addition to dorsal skin damage, repeated DNCB application to mice ears induced swelling and AD-like symptoms, reflecting localized allergic inflammation. SME treatment significantly alleviated ear swelling and reduced inflammatory symptoms after the final treatment (Figure 4D). These findings suggest that SME effectively reduces AD-related skin damage and inflammation, demonstrating its potential as a natural therapeutic agent for AD.

### 3.5. SME Reduced Skin Thickness, Spleen Enlargement, and Serum IgE Levels in AD-like Model

**Figure 5:**
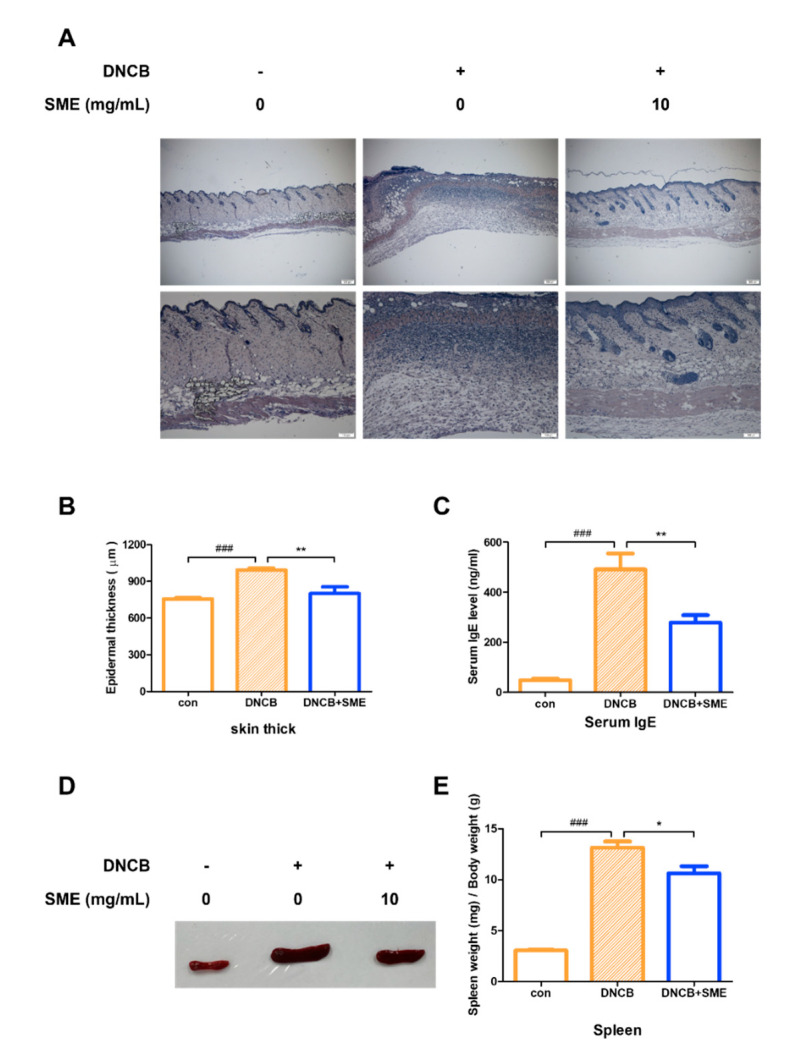
SME reduces skin thickness, spleen enlargement, and serum IgE levels in AD-like model. Hematoxylin and eosin staining analysis of each group. Histological analysis was performed using light microscopy at 40× and 100× magnification (**A**) and thickness was measured using a micrometer (**B**). Data are expressed as the mean ±SD (n = 5). ^###^  *p* < 0.001 and ** *p* < 0.01. Blood was collected from the heart and serum was collected via centrifugation. Serum IgE levels were determined using the Mouse IgE Kit (**C**). Data are expressed as the mean ± SD (n = 4). ^###^  *p* < 0.001 and ** *p* < 0.01. Isolating the spleen (**D**) and measuring the weight of spleen relative to the weight of the mouse (**E**). Data are expressed as the mean ± SD (n = 4). ^###^  *p* < 0.001 and * *p* < 0.05.

Histological analysis of the dorsal skin of the AD-like mouse model revealed that SME treatment significantly alleviated keratinization, reducing skin thickening caused by excessive keratinocyte proliferation (Figure 5A,B). AD is primarily driven by a Th2-dominant immune response, characterized by elevated serum IgE levels, eosinophilia, and Th2 cytokine production. As serum IgE levels are a key marker of AD severity, IgE levels in an AD-like model were analyzed. Our results demonstrated that SME treatment significantly reduced serum IgE levels, suggesting that SME modulates Th2-mediated allergic responses (Figure 5C). Chronic allergic inflammation leads to spleen enlargement, a common indicator of systemic immune activation. In this study, spleen hypertrophy was significantly suppressed in the SME-treated group, further supporting the immunomodulatory effects of SME in AD (Figure 5D,E). These findings suggest that SME alleviates AD-associated skin thickening and reduces systemic allergic responses by lowering IgE levels and preventing immune organ enlargement.

## 4. Discussion

Allergy, classified as type I hypersensitivity, is a critical immune dysfunction that affects individuals globally [28]. Allergic reactions can be triggered by allergens, including food, pollen, dust, cosmetics, mold spores, and animal dander [29]. Upon allergen exposure, an early-phase allergic response occurs within minutes, followed by a late-phase reaction characterized by the release of pro-inflammatory cytokines (TNF-α and IL-4) several hours later [30]. The activation of mast cells and basophils, leading to histamine release, is a key event in allergic reactions. Histamine induces smooth muscle contraction, increased vascular permeability, and vasodilation, ultimately contributing to allergic symptoms [31,32]. Antihistamines are commonly used as a standard anti-allergic treatment. However, despite their efficacy, antihistamines can cause side effects such as cardiotoxicity, central nervous system depression, and anticholinergic effects in certain patients [33].

Considering the limitations of current antihistamines, this study investigated the use of *S. miltiorrhiza* as a novel natural anti-allergic agent. *S. miltiorrhiza* contains major active components, such as tanshinones and salvianolic acids, known for their antioxidant and anti-inflammatory properties, and also possesses antibacterial, antiviral, and anticancer properties, playing a therapeutic role in cardiovascular diseases [34]. Its well-documented safety profile makes SME a promising alternative for allergy treatment.

This study first assessed the inhibitory effects of SME in vitro, focusing on its ability to suppress mast cell degranulation using the RBL-2H3 cell model. Mast cells are the primary effector cells responsible for immediate hypersensitivity reactions in humans, and their activation results in the release of histamine, triggering allergic symptoms [35]. Degranulation is initiated when IgE-bound FcɛRI receptors aggregate upon allergen exposure, leading to mast cell activation [36]. This intracellular signaling cascade is crucial for the propagation of allergic inflammation [37].

Mast cell activation is central to IgE/antigen-mediated immune responses, releasing inflammatory mediators that drive allergic reactions [38]. This study confirmed that SME treatment effectively inhibited mast cell degranulation by reducing β-hexosaminidase release (Figure 1). To further investigate the anti-allergic potential of SME, its effect on anaphylaxis, a severe and acute allergic reaction, was evaluated. Anaphylaxis results from the rapid release of mediators from mast cells and basophils into the bloodstream, leading to life-threatening symptoms, such as laryngeal edema, respiratory failure, and circulatory collapse [39,40]. As the PCA model is widely used to assess anti-allergic activity in vivo, this model was used to evaluate the therapeutic effects of SME. Our results demonstrated that SME treatment significantly reduced Evans blue extravasation, indicating that SME effectively suppressed vascular permeability and mitigated anaphylactic symptoms (Figure 2B,C). To elucidate the molecular mechanisms underlying the anti-allergic effects of SME, we examined its effect on Syk phosphorylation, a key regulator of mast cell activation. Syk is a crucial tyrosine kinase that activates downstream signaling molecules [18,24], thereby controlling mast cell degranulation and inflammatory mediator production [41]. Our findings revealed that SME markedly inhibited Syk phosphorylation (Figure 3A), suggesting that SME disrupted the upstream signaling cascade involved in mast cell activation. Furthermore, we assessed the effects of SME on the mitogen-activated protein kinase (MAPK) pathway, which promotes allergic signaling. SME treatment significantly reduced the phosphorylation levels of AKT, ERK, and JNK, key MAPK pathway components (Figure 3B–D). Since MAPK signaling drives histamine release and cytokine production, these results indicate that SME suppresses allergic inflammation by inhibiting histamine release after allergic responses and targeting Syk-mediated upstream signaling pathways to block the initiation of allergic responses. Overall, the molecular effects of SME confirm that it inhibits allergic responses by blocking key intracellular signaling pathways.

AD is a chronic cutaneous disorder characterized by cellular and humoral immune abnormalities [42]. Patients with AD typically exhibit elevated serum IgE levels, peripheral eosinophilia, impaired cellular immunity, and increased IL-4 production, all contributing to disease pathogenesis [43]. To evaluate the therapeutic potential of SME, a DNCB-induced AD-like mouse model was used to examine clinical symptoms such as erythema, pruritus, exudation, scratch-induced erosion, and lichenification. SME treatment effectively alleviated these symptoms, suggesting its ability to suppress inflammatory skin responses (Figure 4B). Furthermore, as DNCB exposure increased, dorsal skin damage rapidly progressed in the AD model. However, the SME-treated group showed noticeable improvement in skin condition from the fifth treatment onward (Figure 4C).

In DNCB-treated mice, allergic ear swelling led to severe erythema. SME treatment significantly reduced ear swelling and restored it to near-normal levels (Figure 4D). Additionally, SME alleviated keratinization-induced epidermal thickening and disrupted cellular architecture, supporting its protective effects against AD-related skin damage (Figure 5A,B).

**Figure 6:**
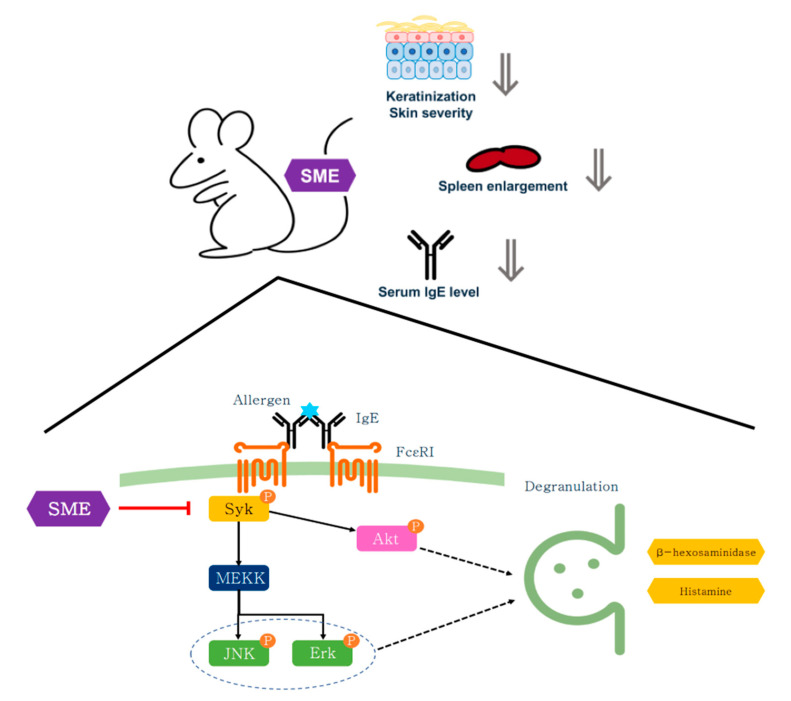
Schematic diagram of anti-allergy activity of SME on mast cell and AD-like model.

Chronic inflammation can often enlarge immune organs, such as the spleen and lymph nodes, due to excessive immune activation [44]. To assess the systemic effects of SME, serum IgE levels and immune organ hypertrophy were analyzed. Blood analysis revealed that SME treatment significantly reduced serum IgE levels compared to the untreated AD group (Figure 5C). Additionally, spleen enlargement, a hallmark of chronic allergic responses, was effectively suppressed in SME-treated mice, as demonstrated by the spleen-to-body weight ratio (Figure 5D,E). This study demonstrates that SME exerts anti-allergic effects across different stages of immune activation, including acute allergic responses (PCA model) and chronic inflammation (AD model). The effectiveness of SME in the cellular assay, the in vivo PCA model, and AD-like mouse model supports its potential as a natural anti-allergic therapy (Figure 6).

Unlike conventional synthetic drugs that target downstream allergic mediators, such as histamine, SME modulates mast cell activation at an upstream level, suppressing both immediate hypersensitivity and chronic allergic inflammation. These findings suggest that SME is a novel mast cell-targeting anti-allergic agent derived from natural sources.

Furthermore, considering its efficacy in both systemic and cutaneous models, SME may be developed into different pharmaceutical formulations depending on the clinical setting. An oral dosage form could be suitable for acute allergic conditions, whereas a topical cream formulation may provide therapeutic benefits for chronic skin inflammation, such as AD. Further research on formulation optimization and pharmacokinetics will help to facilitate its clinical translation.

## Data Availability

The data supporting the findings of this study are available from the corresponding author upon request.

## Supplementary Materials

The following supporting information can be downloaded at: https://www.mdpi.com/article/10.3390/biomedicines13071547/s1, Figure S1. Cytotoxicity of SME toward RBL-2H3 cells. RBL-2H3 cells were treated with SME (50 and 100 µg/mL) for 24 h. Cytotoxicity was measured using the Quanti-Max WST-8 cell viability kit. Absorbance was measured at 450 nm using a microplate reader. Data are representative of at least three independent experiments with similar results. Data are expressed as the mean ± SEM (n = 4).

## Abbreviations

The following abbreviations are used in this manuscript:

AD	atopic dermatitis
DNCB	2,4-dinitrochlorobenzene
DPH	diphenhydramine
FcɛRI	high-affinity IgE receptors
FcRs	Fc receptors
PCA	passive cutaneous anaphylaxis
SME	*Salvia miltiorrhiza* extract
Syk	spleen tyrosine kinase
