# Supplementary material for: Salvia miltiorrhiza Root Extract as a Potential Therapeutic Agent for IgE/Ag-Induced Allergic Reactions and Atopic Dermatitis via the Syk/MAPK Pathway
Source: Biomedicines. 2025 Jun 25;13(7):1547. doi: 10.3390/biomedicines13071547 (PMC12292856; doi:10.3390/biomedicines13071547)
Supplement: Supplementary file 1 [file biomedicines-13-01547-s001.zip › biomedicines-3665616-supplementary.pdf]

biomedicines

Supplementary

Salvia miltiorrhiza Root Extract as a Potential Therapeutic Agent for IgE/Ag-Induced Allergic Reactions and Atopic Dermatitis via the Syk/MAPK Pathway

Min-ah Kim [1], Jin-Ho Lee[1], Keunjung Woo [1], Eunwoo Jeong [1], and Tack-Joong Kim [1,2,*]

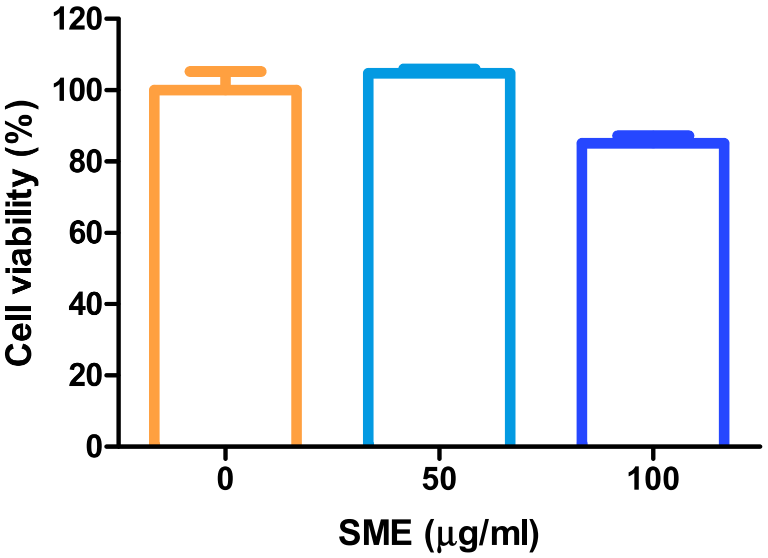

Figure S1. Cytotoxicity of SME on RBL-2H3 cells. RBL-2H3 cells were treated with SME (50 and 100 μg/mL) for 24 h. Cytotoxicity was measured by Quanti-Max WST-8 cell viability kit. Absorbance was measured at 450 nm using a microplate reader. Data are representative of at least three independent experiments with similar results. Data are expressed as mean ± SEM (n = 4).

https://doi.org/10.3390/xxxxx
